# The Impact of Pandemic on Couples With Cancer: Examining the Role of Cancer-Related Communication on Cancer-Related Distress Via the Actor–Partner Interdependence Model

**DOI:** 10.1177/10664807211009803

**Published:** 2022-04

**Authors:** Afarin Rajaei

**Affiliations:** 1 Couples and Family Therapy Program, California School of Professional Psychology, Alliant International University, San Diego, CA, USA

**Keywords:** couples, cancer, dyadic study, APIM, COVID-19

## Abstract

The present study draws attention to the significance of considering cancer-related communication on cancer-related distress through the vulnerability–stress–adaptation model among couples with cancer during the pandemic. This is a quantitative dyadic study with a sample of 80 couples (*N* = 160). Dyadic data were analyzed among couples with cancer to examine the within-person (actor effects) and between-partner (partner effects) associations among links between cancer-related communication and cancer-related distress through the use of actor–partner interdependence models. Significant actor and partner effects were found for cancer-related communication in partners facing cancer, a factor that predicted cancer-related distress. The findings underscore the need to adopt a systemic perspective that accounts for multiple, simultaneous adaptive processes including cancer-related communication as influences on cancer-related distress in the time of COVID-19.

With social distancing becoming the new normal, many couples are getting stuck at home together and their routines got disrupted. This can get worse for couples dealing with preexisting conditions such as cancer. Patients with cancer may be vulnerable to COVID-19 due to their immunecompromised systems. Furthermore, disruptions to treatment sessions due to the stay-at-home orders may add even more distress to couples with cancer and their relationship (Tatrow & Montgomery, 2006). The stress of living through a global pandemic with all its ambiguity and challenges (e.g., financial issues, role changes, social distancing) in addition to challenges associated with a cancer diagnosis (i.e., cancer-related distress) have tension couples’ relationships more than usual. Scholars have explored various adaptive processes (e.g., cancer-related communication) to alleviate cancer-related distress in couples with cancer (Manne & Badr, 2010). But a question remains about the effect of such factors during the pandemic. Thus, in the current study, the role of cancer-related communication on cancer-related distress was examined by vulnerability–stress–adaptation (VSA) model (Karney & Bradbury, 1995) among couples with cancer in the United States during the time of COVID-19.

## Literature Review

Cancer is a chronic illness characterized by the growth of mutated cells in the body (American Cancer Society, 2018). Cancer affects not only individuals but also family members, most notably romantic partners (Tatrow & Montgomery, 2006). Therefore, it is crucial for scholars to have a better understanding of cancer and its impact on individuals, couples, and families.

While cancer has become a public issue in the United States, the struggles of couples coping with cancer often remain private challenges that affect their romantic relationship, physical health, and mental health well-being. While the trials of patients with cancer are well represented in cancer literature (Kornblith et al., 2003), there has been less of a focus on the impact of cancer on romantic relationship rather than just individuals (Acquati & Kayser, 2019). Furthermore, while extant literature points out potential interventions and treatments for patients with cancer, there is a paucity of literature addressing helpful suggestions specifically designed for their cancer-related distress from a dyadic perspective (Badr & Krebs, 2013).

Unlike some other chronic illnesses, the progression of cancer and its consequences (i.e., pain, surgery, financial burdens, and possibly death) is rapid and does not allow partners to prepare for the changes (e.g., partners’ roles, relationship satisfaction, physical and mental well-being on a daily basis) in their lives (Brosseau et al., 2011). This highlights the importance of couples with cancer coping and adaptive processes in their relationship dynamics. Moreover, the pandemic and additional stress factor related to the COVID-19 have made relationships and life more challenging for couples with cancer (e.g., pay cuts and job losses, disruption of health care services). Therefore, the impact of adaptive processes (i.e., cancer-related communication) on cancer-related distress (Manne & Badr, 2010) in the time of pandemic among couples with cancer merits attention.

### Cancer-Related Distress

Receiving the news of a cancer diagnosis is a highly stressful event (NCCN, 2016). It leads to various changes including reorganized priorities, altered schedules, and expectations (e.g., emotional concerns and existential issues, medical treatment and its side effects, an altered sexual image, for example, in breast cancer, and changed social relationships and roles such as caregiver roles; Fletcher et al., 2008). Moreover, adequate and timely recognition and management of cancer-related distress can lead to improved communication and decreased health care utilization (Bidstrup et al., 2011). Therefore, the identification and evaluation of cancer-related distress and its connection to cancer-related communication in the context of a dyadic study during the COVID-19 are essential for health care professions and researchers.

Furthermore, couples cope with cancer and cancer-related distress by enacting a host of diverse adaptive processes (Badr & Krebs, 2013; Regan et al., 2015). Effectively utilizing adaptive processes is linked with numerous positive outcomes including relationship satisfaction and support and improved physical and mental health (Hagedoorn et al., 2011). In light of these facts, a specific adaptive process, which is the cancer-related communication, appears especially pertinent (Manne & Badr, 2010). Although there is some evidence linking cancer-related communication (Manne & Badr, 2010) to cancer-related distress in couples with cancer, the role of this adaptive process on cancer-related distress during the pandemic has gone unexamined.

### Cancer-Related Communication

The way in which couples communicate and how partners communicate work through arguments about their cancer diagnosis is of primary importance (Manne & Badr, 2010). The way partners communicate about cancer-related challenges and stressors including cancer progression and personal and relational priorities may impact the quality of couples’ relationship and the level of cancer-related distress (Acquati & Kayser, 2019; Badr & Carmack, 2008; Manne & Badr, 2008). For example, the frequency of cancer-related conversations, the level of empathy or blame communicated, and the level of compromise around cancer-related challenges or disagreements have been significantly linked with relationship outcomes (Acquati & Kayser, 2019; Badr & Carmack, 2008; Manne & Badr, 2008). Therefore, we investigated the link between cancer-related communication and its potential impact on cancer-related distress during the time of COVID-19 among couples with cancer.

## The Current Study

Given the unparalleled growth in the number of couples with cancer in the United States (American Cancer Society, 2019), it is critical to identify and understand processes influencing cancer-related distress among couples with cancer during the pandemic. In light of the influence of the adaptive processes on cancer-related distress, examining the impact of cancer-related communication on cancer-related distress appears warranted. Against this backdrop, it was aimed to answer the following questions: “How is the cancer-related distress associated with cancer-related distress through the vulnerability–stress–adaptation (VSA) model (Karney & Bradbury, 1995) on both partners involved in couples with cancer during the time of COVID-19?” It was hypothesized both actor and partner effects reflecting the following: Greater cancer-related communication would be associated with lower cancer-related distress.

### VSA Model

**Figure 1.**
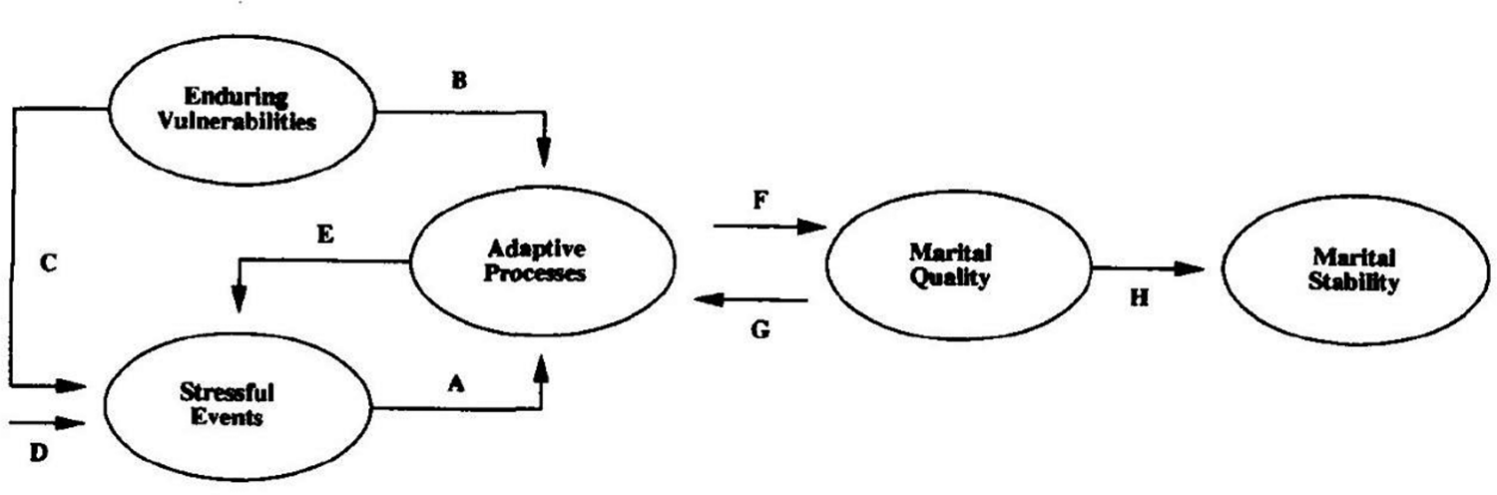
Vulnerability–stress–adaptation model.

The VSA model (Karney & Bradbury, 1995) of marriage explores the manner in which *enduring vulnerabilities*, *stressful events*, and *adaptive processes* often contribute to *marital quality* and *stability* (see Figure 1). Karney and Bradbury (1995) posited that enduring vulnerabilities and stressful events may have significant impacts on romantic functioning and stability, which highlights the importance of the model. Against this backdrop, the VSA model seems a good fit for the population of couples with cancer since it captures the dyadic data in the context of the romantic relationship. In this study, the stressful event is cancer-related distress and pandemic and the adaptive process is cancer-related communication.

## Method

This cross-sectional survey study was designed to extend previous research on cancer-related communication and cancer-related distress in couples with cancer during the pandemic. Dyadic data were analyzed among couples with cancer to examine the within-person (actor effects) and between-partner (partner effects) associations among links between on cancer-related communication and cancer-related distress through the use of actor–partner interdependence models (APIMs; Kashy & Kenny, 2000).

### Participants

All participants, both the partners with cancer and those who were in a relationship with them, were recruited using Qualtrics Panel, an online survey panel service. Qualtrics Panel is an online research company that helps researchers have access to nationwide samples. Couples with cancer were included in the study if (1) both partners were 18 years or older, (2) one partner was diagnosed with cancer, (3) both partners were fluent in reading and writing English, and (4) partners had been in a committed relationship with one another for a minimum of 6 months before participating in the study. Based on estimates from a web-based calculator designed for APIM (Ackerman & Kenny, 2016), the targeted sample size was 80 couples (i.e., *N* = 160).

### Recruitment

After receiving the Institutional Review Board approval, the target sample was recruited via Qualtrics Panel to participate. The couple as a unit received U.S. $15 as an incentive for their participation after each partner of the romantic dyad completed the survey. It took approximately 10 min for each partner/participant to fill out the survey. All measures were completed via a self-reported online survey and are described below.

### Measures

Participants first completed demographic information including age, gender, race, ethnicity, yearly income, level of education, months in a committed relationship with the current partner, relationship status, type of cancer, stage of cancer, type of treatment, and whether they had had surgery or not. Following the completion of the demographics portion of the survey, participants completed the measures described below.

#### Cancer-related distress

Cancer-related distress was captured using the eight-item adapted version of the Impact of Events Scale (Weiss & Marmar, 1997). This scale has been found to reliably assess intrusive thoughts about stressful events and conscious avoidance of feelings and ideas about the events. Internal consistency for the shortened version is as acceptable as the more extended version. Participants were instructed to indicate how often they have been bothered by various distress symptoms (e.g., “I had trouble concentrating”) during the past week. Response options for each item include 1 (*not at all*), 2 (*a little bit*), 3 (*moderately*), 4 (*quite a bit*), and 5 (*extremely*). Scores are summed and range from 8 to 40, with greater scores indicating a higher level of distress and anxiety about the cancer diagnosis in a romantic relationship (Weiss & Marmar, 1997). This scale demonstrated good reliability overall (Cronbach’s α = .74 for patients with cancer and Cronbach’s α = .72 for their partners).

#### Cancer-related communication

The Communication Patterns Questionnaire (Sulmasy, 2002) was used to capture cancer-related communication. We adapted the questionnaire to be cancer-specific by asking the participant to rate how the couple typically deals with cancer-related stressors or problems (e.g., “Cancer or its treatment have been interfering with the leisure or social activities you and your partner usually engage in”). Patients and partners rated their perceptions of their relationship communication when issues arise, during a discussion about the issue, and after the discussion. All items were rated on a 9-point Likert-type scale that ranged from 1 (*unlikely*) to 9 (*likely*), with higher scores suggesting healthier communication about the cancer diagnosis in a romantic relationship (Mann & Badr, 2008). The cancer-related communication scale demonstrated good reliability overall (Cronbach’s α = .75 for patients with cancer and Cronbach’s α = .71 for their partners).

### Data Analysis Plan

Descriptive statistics and intercorrelations between the study variables were first examined. Prior to fitting the models to the data, it was ensured that the data met the assumptions of linear regression. Following linear regression modeling, we conducted APIM (Kashy & Kenny, 2000) in order to analyze the impact of participants’ self-reported independent variables on self-reported (i.e., actor effect) and partner-reported (i.e., partner effect) dependent variables. Dyadic data were gathered for each variable in the hypothesized model (i.e., demographic variables, cancer-related stress, cancer-related communication). The hypothesized APIM model (Kenny et al., 2006) including the independent variable (i.e., cancer-related communication) and the dependent variables (i.e., cancer-related distress) were tested using Mplus software (Muthén & Muthén, 1998–2019). When conducting the analyses and reporting the results, Partner 1 represented the patient with cancer and Partner 2 represented the other romantic partner.

## Results

Table 1 shows demographic information and Table 2 presents descriptive statistics and intercorrelations among all study variables. Separate multiple regression analyses for cancer patients and their partners were conducted. Before conducting multiple regression, the variables were checked to make sure they satisfy multiple regression assumptions including linearity, normality, and homoscedasticity (Gavin, 2008). Histogram examination suggested that none of the variables contained system missing or extreme values. Based on scatter plots of residuals, the assumption of homoscedasticity was verified. The assumption of linearity was then tested through a Q-Q plot, showing that the sample distribution was close to normal.

**Table 1.** Demographic Information.

Characteristics	Partner Facing Cancer (*n* = 80)	Romantic Partner (*n* = 80)
Age		
18–24	1 (1.3%)	1 (1.3%)
25–34	8 (10%)	7 (8.8%)
35–44	10 (12.5%)	8 (10%)
45–54	8 (10%)	12 (15%)
55–64	24 (30%)	22 (27.5%)
65–74	21 (26.3%)	21 (26.3%)
75–84	8 (10%)	9 (11.2%)
Gender		
Male	50 (62.5%)	31 (38.8%)
Female	30 (37.5%)	49 (61.3%)
Ethnicity		
Black	1 (1.3%)	2 (2.5%)
White	71 (88.8%)	65 (81.3%)
Hispanic	8 (10%)	11 (13.8%)
Native American	0	1 (1.3%)
Asian	0	1 (1.3%)
Level of education		
Highschool graduate	10 (12.5%)	19 (23.8%)
Some college	15 (18.8%)	15 (18.8%)
Bachelors’ degree	32 (40%)	22 (27.5%)
Masters’ degree	17 (21.2%)	18 (22.5%)
Doctorate degree	6 (7.5%)	3 (3.8%)
Financial situation		
Extremely difficult	33 (41.3%)	34 (42.5%)
Somewhat difficult	8 (10%)	7 (8.8%)
No difficulty	31 (38.8%)	29 (36.3%)
Not a concern	8 (10%)	10 (12.5%)
Employment status		
Employed full time	32 (40%)	31 (38.8%)
Employed part time	3 (3.8%)	5 (6.3%)
Unemployed and looking for work	1 (1.3%)	0
Unemployed and not looking for work	0	0
Homemaker	4 (5%)	5 (6.3%)
Student	1 (1.3%)	1 (1.3%)
Retired	29 (36.3)	30 (37.5%)
Self-employed	5 (6.3)	5 (6.3%)
Unable to work	5 (6.3%)	3 (3.8%)
Insurance status		
Insured	76 (95%)	75 (93.8%)
Uninsured	4 (5%)	5 (6.3%)
Time in a committed relationship		
Almost 6 months	2 (2.5%)	1 (1.3%)
6 Months to 2 years	1 (1.3%)	3 (3.8%)
2 Years to 5 years	5 (6.3%)	3 (3.8%)
More than 5 years	72 (90%)	73 (91.3%)
Status of relationship		
Committed relationship	5 (6.3%)	4 (5%)
Married	75 (93.8%)	76 (95%)
Partners live together		
No	1 (1.3%)	1 (1.3%)
Yes	79 (98.88%)	79 (98.88%)
Children		
Yes	62 (77.5%)	62 (77.5%)
No	18 (22.5%)	18 (22.5%)
Religious affiliation		
Yes	53 (66.3%)	54 (67.5%)
No	27 (33.8%)	26 (32.5%)
Time of diagnosis		
Less than 3 months ago	1 (1.3%)	1 (1.3%)
Between 3 to 6 months ago	3 (3.8%)	3 (3.8%)
Between 6 to 12 months ago	10 (12.5%)	10 (12.5%)
Between 1 to 3 years ago	19 (23.8%)	19 (23.8%)
More than 3 years ago	47 (58.8%)	47 (58.8%)
Type of cancer		
Breast cancer	28 (35%)	28 (35%)
Skin cancer	4 (5%)	4 (5%)
Prostate cancer	8 (10%)	8 (10%)
Uterine cancer	2 (2.5%)	2 (2.5%)
All others	38 (47.5%)	38 (47.5%)
Stage of cancer		
1	20 (25%)	20 (25%)
2	28 (35%)	28 (35%)
3	20 (25%)	20 (25%)
4	12 (15%)	12 (15%)
Other partner had cancer before		
Yes	65 (81.3%)	15 (18.8%)
No	15 (18.8%)	65 (81.3%)

**Table 2.** Correlations Among Variables.

	*M* (*SD*)	1	2	3	4
1. Partner 1: cancer distress	17.21 (8.34)	1	−.41**	.62**	−.40**
2. Partner 1: Cancer-related communication	49.51 (10.90)		1	−.26*	.79**
3. Partner 2: cancer distress	15.08 (7.60)			1	−.29*
4. Partner 2: Cancer-related communication	51.80 (13.09)				1

*Note*. *M* = mean; *SD* = standard deviation.

*p ≤ .05. **p < .01.

**Figure 2.**
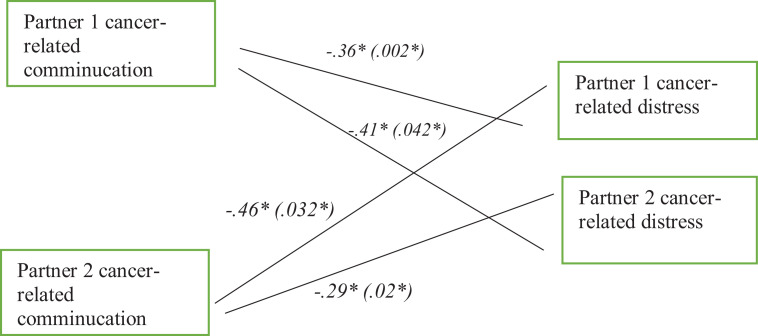
The fully saturated actor–partner interdependence model of cancer-related communication in couples with cancer predicting partners’ cancer-related distress. *Note.* Values for significant pathways were provided. For simplicity, the correlations between the independent variables across the two partners in the figure were not shown but provided them in Table 2. Standardized path coefficients were presented with unstandardized coefficients in parentheses.

After examining multivariate analyses via linear regression models, we next conducted APIMs to simultaneously consider the impact of both actor and partner effects on cancer-related distress for both cancer patients and their partners. The APIM analysis estimated actor and partner effects for cancer-related communication in Partners 1 and Partners 2 on both partners’ cancer-related distress (see Figure 2).

Figure 1 shows the associations between the cancer-related communication for both partners and each partner’s cancer-related distress, controlling for education and income level. We noted negative actor effects for Partner 1 and 2 cancer-related communication and their own cancer-related distress (β = −.36, *p* = .002; β = −.29, *p* = .02). Thus, partners facing cancer who reported greater cancer-related communication also reported a lower level of cancer distress. Additionally, we discovered that greater cancer-related communication as reported by Partner 1 or 2 had negative partner effects. Greater cancer-related communication reported by Partner 1 or 2 was significantly, negatively linked with their partners’ reports of lower level of cancer distress (β = −.41, *p* = .042; β= −.46, *p* = .032). Therefore, when partners of cancer patients or patients themselves reported greater cancer-related communication, their partners reported a lower level of cancer distress during the pandemic time. This model accounted for 58.4% of the variance of cancer-related distress in Partner 1 and 46.1% of the variance cancer-related distress in Partner 2.

## Discussion

Although the importance of adaptive processes such as cancer-related communication for couples facing cancer and their cancer-related distress and romantic relationships have been highlighted in previous studies (Zeidner et al., 2013), there is a lack of research considering the effects of different types of adaptive processes (e.g., cancer-related communication; Hagedoorn et al., 2000) during the time of COVID-19. Thus, there exists a need for the simultaneous examination of associations among adaptive processes and cancer-related distress for couples with cancer during the pandemic.

To this end, the current study extended the literature by examining how cancer-related distress as an adaptive process may be associated with cancer-related distress in couples with cancer during the pandemic. Data analysis partially supported the hypotheses mentioned above. Significant actor and partner effects were found for cancer-related communication in partners facing cancer, a factor that predicted cancer-related distress. The findings underscore the need to adopt a systemic perspective that accounts for multiple, simultaneous adaptive processes including cancer-related communication as influences on cancer-related distress in the time of COVID-19.

### Cancer-Related Communication and Cancer-Related Distress During the Pandemic

The noteworthy findings from this study were that cancer-related communication in couples facing cancer had both actor and partner effects on cancer-related distress. In line with Badr and Krebs (2013), cancer-related communication may have significant implications for couples including self-efficacy, perceptions of intimacy, psychological health outcomes, and relationship stability. It is possible that cancer-related distress was affected by the additional anxiety and frustration brought on by the pandemic. COVID-19 has inevitably disrupted many couples’ established routines (e.g., partner’s roles, relationship rituals, physical and mental self-care strategies on a daily basis), and these disruptions are even more debilitating for couples dealing with preexisting conditions such as cancer. In addition to their immunecompromised systems that enable patients with cancer to be at greater risk for contracting COVID-19 (American Cancer Society, 2019), disruptions to treatment sessions due to the stay-at-home orders may add even more distress to patients with cancer and their partners, perhaps notably altering the couple’s cancer-related distress and cancer-related communication.

Previous research has shown that not all communication about cancer is helpful, and specific types of communication (e.g., urging partners with cancer to increase healthy behaviors) have been associated with lower levels of physical and mental health (Manne et al., 2006).On the other hand, communication of love and gratitude were associated with higher levels of quality of life and relationships in partners with cancer. It is also possible that partners and the relationship were saturated with conversations and logistical planning for dealing with the worldwide pandemic, and therefore, cancer-related communication impacted cancer-related distress lower than usual. In fact, patients with cancer do not live in vacuums, rather, they are inextricably embedded in contexts. Against this backdrop, COVID-19 is a critical context to be considered in future work examining couples with cancer. Qualitative research could explore the dynamics operating in these couples, providing useful insights into partners’ experiences and how each partner’s cognitive and emotional responses to cancer-related communication are perceived by the other person.

It is worth mentioning that no published study has examined the impact of cancer-related communication on cancer-related distress in couples with cancer from dyadic data and through APIM analysis and during the pandemic. Thus, the significant finding of this study needs to be viewed with caution until further research is carried out. Nonetheless, this result provides unique contributions in terms of highlighting the crucial role of cancer-related communication for partners with cancer. This suggests that the less cancer-related communication in partners with cancer may be connected to reports of higher cancer-related distress from their partners. It is also possible that this result indicates the need for interventions aimed at increasing cancer-related communication in patients with cancer to decrease cancer-related distress. Oncology literature suggests that higher levels of dyadic cancer-related distress were associated with higher levels of problematic health issues for the caregiver, anxiety and depression, relationship dissatisfaction, lack of family support, and caregiving burden (Baucom et al., 2019; Milbury et al., 2013). Distress is best studied by assessing data from both members of the dyad (Kenny et al., 2006).

## Strengths and Limitations

The current study provides unique contributions to understanding the association between cancer-related communication and cancer-related distress in couples with cancer during the pandemic. First, this study is the first to recognize the effects of the adaptive processes between cancer-related communication and cancer-related distress in couples with cancer in the time of COVID-19. Second, simultaneously testing the effects of the adaptive process and cancer-related distress informed by the sound theoretical grounding of the VSA model allowed for a deeper understanding of multidimensional factors contributing to cancer-related distress in couples with cancer during the pandemic. Finally, the current study, unlike the other studies, was conducted with dyadic data, collected data from a nationwide sample and utilized measures and the APIM model that assess and analyze relationship quality and stability within a romantic relationship context by taking its interpersonal nature into account without limiting the sample to married or heterosexual couples. Overall, these findings yielded important results in terms of conceptualizing cancer-related distress in couples with cancer during the pandemic.

However, as with all research, the current study had several limitations. It is difficult to generalize study findings due to the following reasons: (a) sample homogeneity (i.e., predominantly White, middle age, educated), (b) methodological limitations, and sample size. Future studies should use mixed methods, qualitatively tapping into meaningful individual experiences, to deeply understand the contexts of couples with cancer. In addition, a larger sample size would promote validity and generalizability. Furthermore, rather than objective observational measures, we utilized self-report surveys, which are always subject to important limitations (e.g., personal biases, inaccurate cognitive recall, social desirability). Lastly, the results must be interpreted cautiously due to the potential limitations of using panel data including interviewer effects, measurement errors, and prestige bias.

## Implications

Patients with cancer often report that their most important relationship is with their caregiver—who is most often their romantic partner (Hagedoorn et al., 2000). Given the importance of this relationship for cancer patients, the findings provide additional support for studying cancer through a dyadic lens rather than as an individual experience. Clinicians should seek to work with both partners and consider both functional and dysfunctional adaptive processes utilized for coping with this strenuous life event. In addition, clinicians should make efforts to incorporate partners of patients with cancer into the treatment plan and recommend therapy sessions regularly. Failing to account for the shared experience of cancer on couples will likely result in ongoing reports of unmet psychosocial needs and heightened distress among patients and their partners. These findings highlight the importance of attending to the mentioned gap and are beneficial to couples and therapists in traditional and integrated behavioral health care (i.e., primary and secondary settings) settings, where there is a limited interaction time with patients. Thus, it is crucial to identify and prioritize which adaptive behaviors (e.g., cancer-related communication) may be useful for couples regarding their cancer-related distress.

This study provides an opportunity for researchers and health care providers to broaden their perspective and consider couples facing cancer from a more comprehensive approach through the VSA model (Karney & Bradbury, 1995). These efforts are necessary in order to understand the complexities of the issues contributing to the relationship quality and stability among couples facing cancer. Patients with cancer do not usually go through cancer in isolation as they are in constant interaction with their support systems (e.g., Rajaei et al., 2020a, 2020b), thus research from a relational and dyadic lens may provide valuable insight into the complexity of couples with cancer lived experiences. Further, the current study and its limitations and strengths may lead researchers to provide essential suggestions and considerations for issues that should be addressed when planning research studies examining couples with cancer during the pandemic (e.g., having a more diverse sample, employing multivariate statistical tests that can capture the complexity of the multidimensional factors affecting the relationship between adaptive processes and cancer-related distress or conducting qualitative studies in order to deepen our understanding of adaptive processes and/or cancer-related distress in couples with cancer).

## Conclusion

Utilizing a dyadic quantitative approach through APIM (Kenny et al., 2006), the current study aimed to capture the associations among links between cancer-related communication and cancer-related distress in couples with cancer in the time of COVID-19. Findings from this study are critical to ensuring psychosocial–spiritual care that will meet the needs of couples with cancer and their cancer-related distress. The findings underscore the importance of assessing both patients’ and partners’ adaptive processes such as cancer-related communication and also cancer-related distress in couples with cancer during the pandemic. Overall, these findings highlight the importance of taking a dyadic perspective when studying the adaptive processes with cancer-related distress in couples with cancer during a stressful event such as pandemic.
